# A High-Fat High-Sucrose Diet Rapidly Alters Muscle Integrity, Inflammation and Gut Microbiota in Male Rats

**DOI:** 10.1038/srep37278

**Published:** 2016-11-17

**Authors:** Kelsey H. Collins, Heather A. Paul, David A. Hart, Raylene A. Reimer, Ian C. Smith, Jaqueline L. Rios, Ruth A. Seerattan, Walter Herzog

**Affiliations:** 1 Human Performance Laboratory, University of Calgary, AB, Canada; 2 McCaig Institute for Bone and Joint Health, University of Calgary, AB, Canada; 3 Department of Biochemistry and Molecular Biology, University of Calgary, AB, Canada; 4 The Centre for Hip Health & Mobility, Department of Family Practice, University of British Columbia, Vancouver, BC, Canada; 5 CAPES Foundation, Brasilia, Brazil

## Abstract

The chronic low-level inflammation associated with obesity is known to deleteriously affect muscle composition. However, the manner in which obesity leads to muscle loss has not been explored in detail or in an integrated manner following a short-term metabolic challenge. In this paper, we evaluated the relationships between compromised muscle integrity, diet, systemic inflammatory mediators, adipose tissue, and gut microbiota in male Sprague-Dawley rats. We show that intramuscular fat, fibrosis, and the number of pro-inflammatory cells increased by 3-days and was sustained across 28-days of high-fat high-sugar feeding compared to control-diet animals. To understand systemic contributors to muscle damage, dynamic changes in gut microbiota and serum inflammatory markers were evaluated. Data from this study links metabolic challenge to persistent compromise in muscle integrity after just 3-days, a finding associated with altered gut microbiota and systemic inflammatory changes. These data contribute to our understanding of early consequences of metabolic challenge on multiple host systems, which are important to understand as obesity treatment options are developed. Therefore, intervention within this early period of metabolic challenge may be critical to mitigate these sustained alterations in muscle integrity.

Muscle mass is considered one of the key indicators of longevity^1^. Clinically, many individuals with obesity also have sarcopenia, or muscle wasting^2^. This muscle wasting includes both intramuscular adipose accumulation and muscle fibrosis^3^, and moreover, intramuscular fat and inflammatory cell accumulation is associated with the onset of insulin resistance^4^. However, efforts to understand early muscular changes associated with high-fat diet in both humans and pre-clinical models have been largely centered around changes in glucose regulation, metabolism, mitochondrial dysfunction, inflammatory cell accumulation^5^,^6^,^7^,^8^,^9^,^10^, and indirect observations of intramuscular lipid deposition^5^. Furthermore, these studies are often conducted after obesity has been induced. Over time, increased intramuscular lipid content leads to decreased muscle protein anabolism and compromised muscle repair, resulting in long term, dynamic, and pronounced decreases in muscle integrity^3^. Although alterations in muscle integrity have been observed after a standard obesity induction period^11^, the manner in which muscle morphological changes occur early in the induction phase of diet-induced obesity remain unclear, as few studies focus on elucidating early atrophy-related changes and aberrant repair processes with metabolic challenge^5^,^12^.

Adipose tissue lipid storage is altered with obesity, and adipose tissue fibrosis is considered a hallmark of metabolic alterations on adipose tissue^3^,^13^. Moreover, insulin resistance is reported to be a consequence of human adipose tissue fibrosis^13^. Cross-talk has been observed between adipose tissue and skeletal muscle during obesity onset^14^. Both mechanically and biologically, infiltration of fat and fibrosis in muscle tissue can compromise function, insulin sensitivity and biological homeostasis within the muscle^3^,^11^. However, the determinants of this intramuscular fibrosis, or whether this fibrosis relates to intramuscular adipose tissue deposits, are unclear, particularly early in the induction phase of diet-induced obesity^3^,^14^.

Furthermore, the gut microbiota may be a potential link between high-fat high-sucrose (HFS) diet, systemic chronic inflammation, and musculoskeletal integrity^15^,^16^,^17^. For example, diet-dependent microbial products and co-metabolites have been suggested to influence metabolism of host tissues including muscle^7^. However, the serial short-term relationships between changes in the gut microbiota, serum inflammatory mediators, visceral adipose tissue inflammation, intramuscular fat deposition, muscular inflammation, and fibrosis, remain to be clarified in a preclinical model of metabolic challenge.

The purpose of the present set of studies was to determine the time-course of vastus lateralis (VL) intramuscular fat accumulation and development of fibrosis, in conjunction with short-term systemic (inflammatory cytokines), tissue (adipose tissue and VL muscle), gut microbial, and molecular alterations (inflammation, oxidative stress) following a short-term HFS metabolic challenge. These data will provide a framework linking system-wide changes early in the inductive phase of obesity that result in compromised muscle integrity^12^.

## Results

### Body Fat Percentage and Body Mass Increase with Short Term HFS Exposure

**Figure 1:**
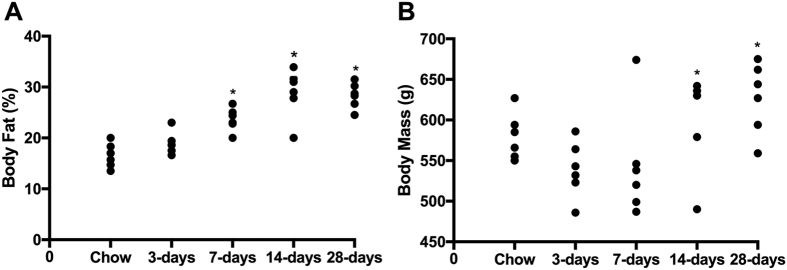
Short-term high-fat high-sucrose metabolic challenge induces alterations in body composition and body mass. (**A**) Body fat increases at 7-days on a high-fat high-sucrose (HFS) metabolic challenge, and is sustained over 28-days of feeding compared to chow-fed controls. (**B**) Body mass, however, doesn’t increase significantly until 14-days on HFS metabolic challenge compared to chow-fed control animals. Raw data are shown, *indicates p ≤ 0.05 compared to chow-fed controls.

Animals fed HFS for 7-days had more body fat (Fig. 1a) than chow-fed control animals, whereas HFS feeding for 14-days and 28-days animals had more body fat and body mass (Fig. 1a,b) compared with chow-fed control animals (p < 0.05).

### Alterations in muscle integrity and inflammatory cell number are detected by 3-days on HFS

**Figure 2:**
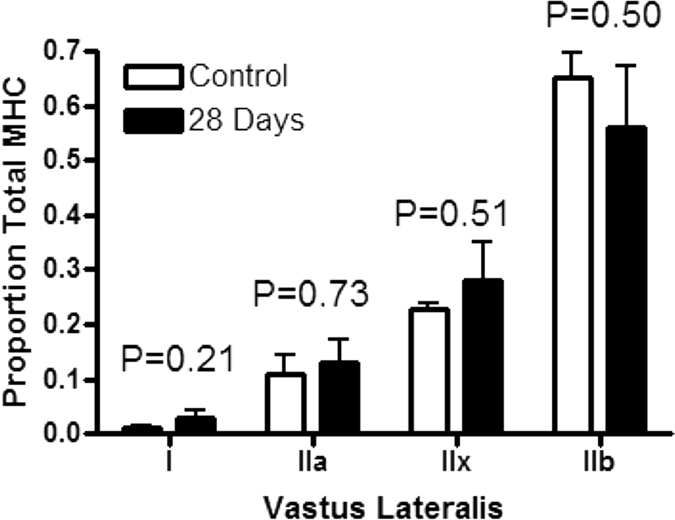
Myosin heavy chain (MHC) distribution in vastus lateralis muscles of chow-fed control rats and rats fed a high-fat high-sucrose diet for 28-days.

**Figure 3:**
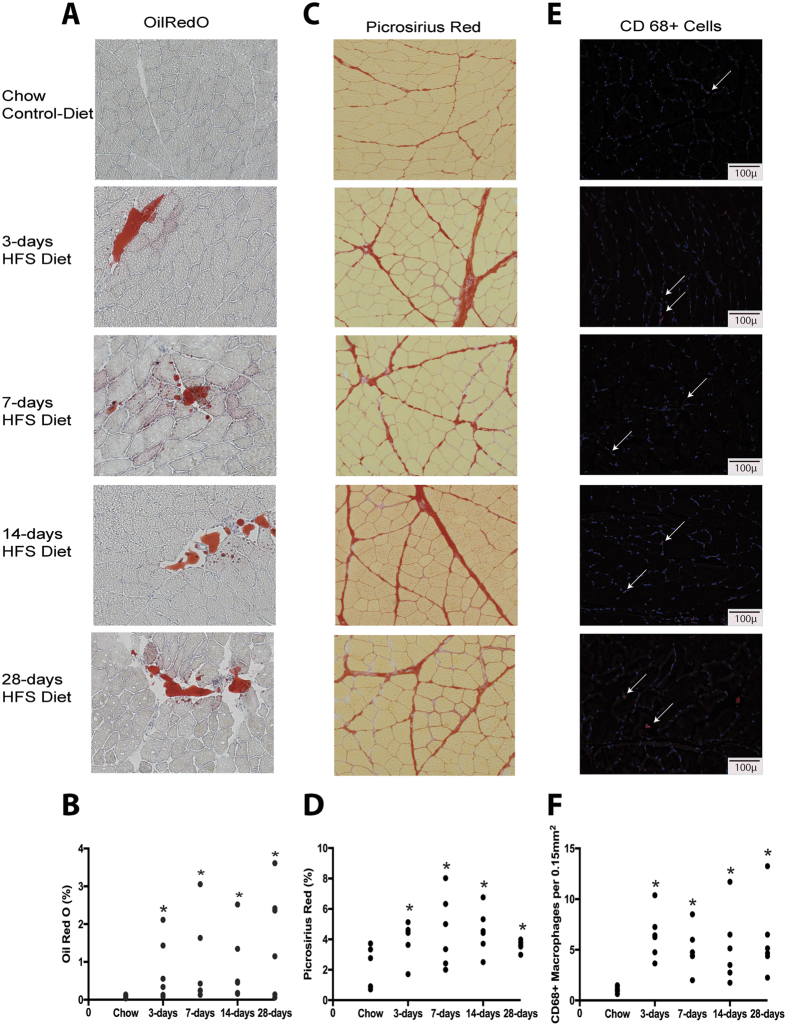
Histological assessments of VL muscle demonstrate rapid changes in muscle integrity with short-term high-fat high-sucrose metabolic challenge. (**A**) Oil Red O Stainining for intramuscular lipid in vastus lateralis (VL) muscle sections taken at 100x magnification. (**B**) Raw average values for Oil Red O Staining for each animal, where 20–50 images were evaluated for a mid-belly cross-section. By 3-days of HFS feeding, animals demonstrated increased and sustained intramuscular lipid staining compared to chow-fed control animals. *indicates p ≤ 0.05 compared to chow-fed controls. (**C**) Picrosirius red staining for collagen in VL muscle sections, imaged at 100x magnification.(**D**) Raw average values for Picrosirius red staining for each animal, where 20–50 images were evaluated for a mid-belly cross-section. By 3-days of HFS feeding, animals demonstrated increased and sustained collagen staining compared to chow-fed control animals. *indicates p ≤ 0.05 compared to chow-fed controls. (**E**) Immunohistochemistry staining for CD68+ cells in VL muscle sections, imaged at 200x magnification.(**D**) Raw average values for CD68+ staining for each animal, where 8–10 images were randomly selected and evaluated for a given mid-belly cross-section. By 3-days of HFS feeding, animals demonstrated increased and sustained CD68+ staining compared to chow-fed control animals. *indicates p ≤ 0.05 compared to chow-fed controls.

Absolute VL mass was similar across time-points (p > 0.05). After 28-days on the HFS diet, rat VL myosin heavy chain (MHC) isoform distribution had not deviated significantly from that of the chow-fed control group (Fig. 2). VL muscles from HFS animals demonstrated significantly increased Oil Red O (ORO) staining, fibrosis, and CD68+ cell staining from 3-days on the diet (Fig. 3), which was sustained through 28-days, when compared with chow-fed control animals (p < 0.05).

### Molecular changes in Visceral Adipose and VL Inflammation and Oxidative Stress suggest Transient Perturbation

Dynamic alterations in visceral adipose tissue iNOS mRNA levels were evident over time, with a significant upregulation by 7-days on the HFS diet, and with trends towards increases at 3-days and 14-days (Table 1). PPARγ and MCP-1 mRNA levels were significantly upregulated after 28-days on the HFS diet.

Fluctuations in VL muscle mRNA levels were also detected (Table 1). iNOS mRNA levels were increased at 3-days and 7-days in HFS animals, but returned to chow-fed control levels by 14-days. Both PPARγ and MuRF-1 mRNA levels were increased at 7- and 28-days, while MCP-1 mRNA levels were increased at 7-days compared to chow-fed control animals with a trend towards an increase at 28-days. Finally, COX-2 mRNA levels were increased at 7-days in HFS animals (p = 0.009). No differences in VL mRNA levels were detected between HFS and chow control-diet animals at 14-days.

### Serum Inflammatory Profiles are altered by 3-days with a HFS challenge

**Figure 4:**
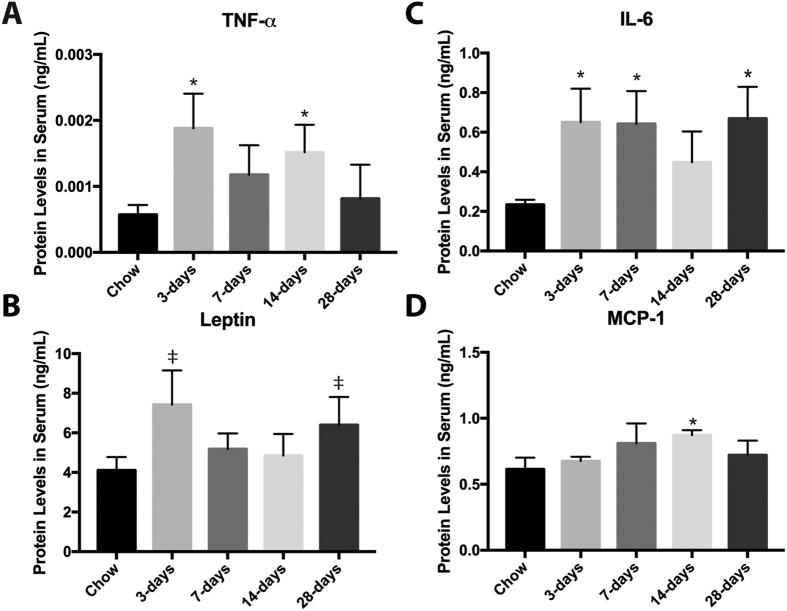
Fluctuations in serum inflammatory mediators with high-fat high-sucrose metabolic challenge suggest dynamic systemic perturbations and restoration of homeostasis. (**A**) Serum TNF-α was increased at 3-days in HFS animals, and then transiently increased again at 14-days on HFS compared to chow-fed control animals. (**B**) Serum leptin demonstrated a trend toward increases at 3-days, and 28-days compared to chow-fed controls. (**C**) IL-6 was increased at 3-days, 7-days, and 28-days in HFS animals compared to chow-fed control animals. (**D**) MCP-1 was increased at 14-days on HFS compared to chow-fed controls. ^‡^indicates p < 0.10 vs chow-fed control; *indicates p ≤ 0.05; data are demonstrated as fold-change compared to chow-fed control and shown as mean ± SE.

Similar levels of blood glucose and insulin were detected between all groups (Table 2). TNF-α and IL-6 increased with the HFS diet as early as 3-days compared to chow-fed controls, while leptin levels demonstrated a trend toward increases in the HFS animals (p = 0.061, Fig. 4). Levels of serum markers then proceeded to fluctuate over the time-points evaluated, such that at 7-days IL-6 levels remained significantly increased (p = 0.05), TNF-α and MCP-1 levels were increased at 14-days (p < 0.05), and at 28-days IL-6 levels were again increased (p = 0.005) while leptin levels exhibited a trend towards elevations (p = 0.10).

### Dynamic Alterations in Gut Microbiota were observed by three days with HFS Challenge

**Figure 5:**
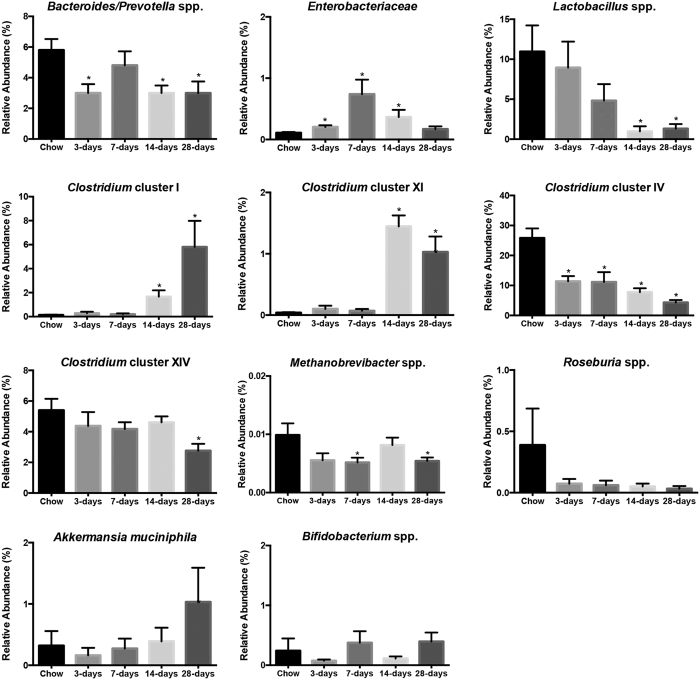
Dynamic alterations in the relative abundance of gut microbial profiles were observed with short term high-fat high-sucrose metabolic challenge. *indicates p ≤ 0.05 compared to chow-fed control diet animals, data are shown as mean ± SE.

**Figure 6:**
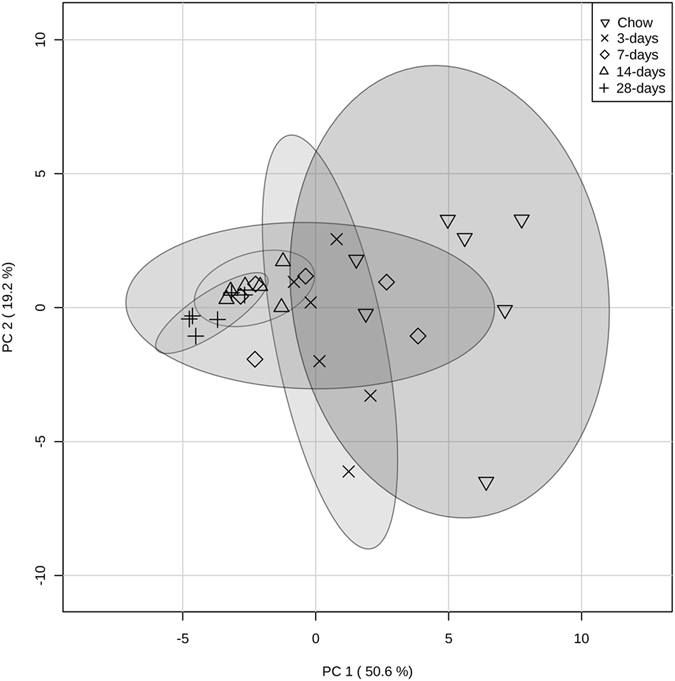
Principal component analysis of the gut microbial relative abundance data reveals tighter clustering of samples with prolonged exposure to high-fat high-sucrose diet. Results are plotted according to the first two principle components, which explain 50.6% (PC1) and 19.2% (PC2) of the variation in gut microbial composition between samples. The clustering of samples within each group is represented by their respective 95% confidence interval ellipse.

Differences in gut microbiota composition between HFS animals and chow-fed controls were evident as early as 3-days exposure to the HFS diet (Fig. 5). Specifically, HFS animals displayed a decreased relative abundance of *Bacteroides/Prevotella* spp., which remained decreased after 14- and 28-days of HFS exposure, and a decreased abundance of *Clostridium* cluster IV compared to chow-fed controls, which was sustained throughout the study. Furthermore, the relative abundance of *Enterobacteriaceae* was increased by 3-days, 7-days, and 14-days of HFS exposure, however returned to levels similar to chow–fed controls by 28-days. Relative abundance of *Lactobacillus* spp., *Clostridium* cluster I, and *Clostridium* cluster XI showed consistent changes within each microbial group in the 14- and 28-day HFS animals compared to chow-fed controls. Moreover, Relative abundance of *Clostridium* cluster XIV was decreased in HFS animals only at 28-days. Finally, the relative abundance of *Methanobrevibacter* spp. was decreased in 7- and 28-day HFS animals. There were no differences detected in abundance of *Bifidobacterium* spp., *Roseburia* spp., and *Akkermansia muciniphila* compared to chow-fed controls. Finally, unsupervised PCA analysis suggested that the overall composition of the gut microbial profiles were most similar in the 14- and 28-day HFS animals (Fig. 6). These data are consistent with the dynamic reshaping of the gut microbiota composition over time in response to overall changes in diet.

### Associations between outcomes suggest an integrated and dynamic biological shift leading to compromised muscle integrity

A positive significant relationship was detected between serum leptin levels and mRNA levels for MAFbx/atrogin-1 (R = 0.40, p = 0.027). VL intramuscular fat, or average percentage of ORO stain, was associated with body fat (R = 0.54, p = 0.002), but did not exhibit significant relationships with any measured serum cytokines or adipokines. Although VL fibrosis, or average percentage of picrosirius red staining, did not demonstrate significant relationships with body fat or serum inflammatory mediators, three intramuscular markers, however, did demonstrate significant positive relationships with fibrosis over all time-points: COX-2, mRNA levels (R = 0.42, p = 0.019), iNOS mRNA levels (R = 0.48, p = 0.007) and CD68+ cell number (R = 0.52, p = 0.003). Furthermore, VL mRNA levels for the fat cell differentiation marker, PPARγ also exhibited a significant positive relationship with VL percent of picrosirius red staining (R = 0.40, p = 0.036). Of note, COX-2 and PPARγ were strongly positively associated with VL iNOS levels (R = 0.80, p < 0.001 and R = 0.84, p < 0.001, respectively).

## Discussion

Muscle integrity is important in obesity, but the development of compromised muscle integrity with obesity is poorly understood. Specifically, the relationships between compromised muscle integrity, systemic mediators, adipose tissue, and gut microbiota, have not been explored in detail or in an integrated manner following a short-term metabolic challenge. Based on the data from this study, we suggest that a link exists between HFS metabolic challenge and persistent increases in intramuscular fat, fibrosis, and CD68+ inflammatory cell number in the VL muscle by 3-days on an HFS diet, although the specific mediator(s) responsible remain to be determined. In contrast to the sustained alterations in the VL muscle, the dynamic alterations observed in local gene expression, systemic inflammation and the gut microbiota early in the obesity induction phase could represent a reciprocal, dynamic competition of perturbations, which ultimately fails. This failure may initiate the process leading to obesity and compromised muscle integrity, potentially setting the stage for which progressive, chronic musculoskeletal alterations with obesity may be established^11^. Although the present study was not designed to evaluate direct causal or mechanistic relationships between metabolic challenge and muscle integrity, the insight gained through this integrated investigation provides critical understanding for the role of obesity on altered muscle integrity.

Abnormal muscle repair is defined by sustained muscle fibrosis, which interferes with the appropriate healing of muscle tissue^18^. Notably, as previous studies have indicated that diet-induced obesity disrupts muscle repair processes^19^, the fibrosis observed in this study may be interpreted as HFS-induced muscle damage or disrupted repair. Here, the increased fibrosis observed by 3-days on the HFS diet is among the earliest reports of compromised muscle integrity with short-term metabolic challenge. Importantly, since fibrosis is a typical step in healthy muscle repair processes, the large variation in fibrosis in 7- and 14-day animals may reflect variation in responses among the animals to the severity of compromise in muscle with the HFS challenge. A tightening of such variation at 28-days suggests that a threshold may have been reached beyond which muscle repair is difficult. Therefore, 28-days on the HFS diet may prove to be an important time-point to determine whether muscle damage may still be reversible^11^.

Furthermore, little is known about how intramuscular fat may influence the muscle repair and regeneration process. However, ectopic fat accumulation in non-adipose tissues is a key feature of metabolic dysregulation^3^ and increased myocellular lipid content leads to increased lipid burden and insulin resistance^3^. In the present study, increased fibrosis at 3-days may result from increases in intramuscular fat deposition, which may signal the infiltration of pro-inflammatory cells, and they become activated. Additionally, VL fibrosis was associated with intramuscular constituents likely related to intramuscular fat (PPARγ) and resulting muscular inflammation (COX-2, iNOS), further implicating a link between intramuscular lipids and fibrosis. However, the present analysis of intramuscular fat did not differentiate between lipid location within the muscle (i.e. intramyofiber lipids, external muscle cell deposits, and non-muscle fat-containing cells) due to the small percentages of intramuscular fat detected in this short time frame, which is a limitation that will be addressed in future studies.

Moreover, dysregulated repair may be indicated by the increased presence of CD68+ cells that occurred by 3-days and was sustained over the 28-day metabolic challenge. MCP-1, a proinflammatory macrophage chemo attractant, was transiently increased in muscle after 7-days on the HFS diet, and despite these transient increases, increases in CD68+ cells compared to chow-fed control values were sustained. Although MCP-1 and macrophages both have known protective and regenerative effects in skeletal muscle^20^, overexpression of MCP-1 coupled with increases in macrophages in skeletal muscle can induce inflammation, lead to alterations in glucose metabolism^5^, are associated with the development of insulin resistance^21^, and may alter intramuscular fat cell function, inducing fat cell differentiation^22^. Although our animals did not display significant differences in fasting glucose or insulin levels at the early time points assessed, these data suggest that dynamic molecular changes in muscle inflammation can lead to sustained muscle damage, potentially before overt metabolic dysregulation is established.

Whether compromised muscle integrity is secondary to the dynamic fluctuations in the microbiota, and subsequent down-stream increases in systemic inflammation, remains to be confirmed. However, we did observe dynamic changes in the gut microbiota composition in HFS-fed animal as early as 3-days, which is consistent with short-term changes observed in both humans^23^ and rodents^24^. Here, it appears that certain microbial groups were more sensitive to the abrupt change in diet, as evidenced by significant deviations from the chow-fed control animals at earlier time-points. However, the gut microbial community is a complex ecosystem, where each microbial taxa may play a different role in establishing its overall composition^25^. Furthermore, studies indicate that while diet can induce rapid changes in gut microbiota composition, these alterations may be transient due to the high resiliency of the gut microbiota community composition, and can thus return to a stable state^26^. These notions are consistent not only with the observed patterns in each microbial group profiled here, but also with the PCA analysis, which indicates that the overall composition of the gut microbiota was most similar in animals exposed to HFS for longer time periods. Altogether, these data suggest that, upon initial exposure to the HFS diet, acute changes in gut microbiota occur, which then ultimately impact the overall composition observed at the 28-day time point.

There is considerable evidence in animal models that the gut microbiota affects host physiology, including muscle function^16^,^27^. Although the specific molecular mechanisms involved in crosstalk across the gut-microbiota-muscle axis^16^,^27^ and the relationship between muscle properties and the gut microbiota remain poorly understood^28^, IL-6, MCP-1 and TNF-α have been identified as potential mediators between the gut microbiota and downstream alterations in muscle function^27^,^29^. Circulating levels of these three pro-inflammatory cytokines, which have been reported to be associated with diet-induced changes in the gut microbiota, are linked with muscle atrophy, lower muscle mass and reduced muscle strength in humans and animals^30^, further linking gut microbiota, systemic inflammation, and compromised muscle integrity in the present study. Specifically, others have shown that high fat diet increases gut permeability, which allows bacterial lipopolysaccharide (LPS) translocation^31^. In turn, LPS binds to toll-like receptor-4, and induces circulating IL-6 and TNF-α^32^, which are reported to be associated with muscle atrophy, lower muscle mass, and reduced muscle strength in humans and animals^30^, and were increased at 3-days in the present study, further linking gut microbiota, systemic inflammation, and compromised muscle integrity in this study. Although we did not directly measure LPS in these animals, we suggest that inflammation resulting from increases in circulating IL-6, and TNF-α, potentially due to diet-induced dynamic changes in gut microbial composition, may recruit pro-inflammatory macrophages to muscle and adipose tissue, ultimately promoting infiltration of macrophages as early as 3-days in the muscle. Notably, the levels of CD68+ cells in VL muscle did not subside, even though both the changes in gut microbial composition, VL and adipose mRNA, and serum inflammatory markers fluctuated throughout the study. Taken together, these data suggest that even short-term exposure to a HFS diet may lead to lasting changes in the VL muscle. Finally, as the mechanistic link between the changes in gut microbiota and systemic alterations detected in the present study may involve increases in gut permeability and subsequent translocation of LPS across the intestinal barrier, these possibilities will be explored in future studies to facilitate a deeper understanding of the short-term changes described here.

In summary, we suggest that short-term metabolic challenge results in rapid compromise of VL muscle integrity and elevation in tissue-level inflammation, resulting from systemic inflammation and alterations in the gut microbiota following short-term exposure to a HFS diet. Obesity induction appears to be a dynamic process that includes compensation across tissues and systems. Intervention or modulation within this early period may be of interest to mitigate important sustained alterations in muscle that occur early in the obesity induction period.

## Methods

Thirty-one male Sprague-Dawley rats were individually housed on a 12/h dark/light cycle, and were allocated to either a high-fat high-sucrose diet group (HFS, 40% fat 45% sucrose; custom Diet #102412, Dyets, Inc, n = 24) or to a control chow-diet (Chow, 12% fat, 3.7% sucrose, n = 7, Lab Diet 5001). All experiments were approved by the University of Calgary Life and Environmental Sciences Animal Care Committee, and all methods were performed in accordance with guidelines and regulations at the University of Calgary. Animals were allocated to one of the following groups: (i) euthanized after 3-days (n = 6); (ii) euthanized after 7-days (n = 6); (iii) euthanized after 14-days (n = 6); (iv) euthanized after 28-days (n = 6); or (v) a chow-fed control group (n = 7). Animal ages at the start of the study and at the time of euthanasia are listed in Table 3.

### Body composition, adipose tissue and muscle characteristics

Animals were euthanized by barbiturate overdose (Euthanyl®, MTC Animal Health Inc., Cambridge, Ontario, Canada). Immediately after sacrifice, body composition was measured using Dual Energy X-ray Absorptiometry and analyzed with software for small animal analysis (Hologic QDR 4500; Hologic, Bedford, MA). Subsequently, VL muscles were weighed and flash-frozen in liquid nitrogen. Visceral adipose tissue was isolated superiorly to the right kidney of each animal and flash-frozen in liquid nitrogen. All tissues were stored at −80 °C until analysis. Myosin heavy chain isoforms (MHC) from VL were separated using SDS-page gel electrophoresis on 4.5% and 7.5% acrylamide stacking and separating gels, according to previously described methods^33^ using a Bio-Rad (USA) Mini-Protean unit (73 V for 40 h). Gels were stained with Coomassie Blue, and imaged on a GS-800 Calibrated Densitometer (Bio-Rad, USA). Lane densities were quantified using the Gel Analysis features of Image J, and the optical densities of the bands corresponding to MHC I, IIa, IIx, and IIb were determined using Fityk curve fitting software.

### Gut Microbiota qPCR analysis

Gut microbiota were measured using qPCR according to previous methods^15^. Standard curves for microbial quantification were normalized to 16S rRNA gene copy numbers obtained from the rrnDB^34^. Copy number of genera averages were used if specific strain information was not available. Relative abundance of each microbial group was reported as percentage of total 16 S rRNA gene copies. Unsupervised clustering of overall gut microbial composition was assessed by principal component analysis (PCA) using MetaboAnalyst 3.0^35^. Relative abundance data was pareto-scaled prior to PCA analysis.

### Serum Marker analysis

Blood serum was collected, prepared, and analyzed for glucose and protein as previously described^15^,^36^. Five markers were quantified in serum using a custom multiplex assay and Luminex®xMAP technology (Eve Technologies, Calgary, AB; Custom Luminex 5-plex: leptin, insulin, MCP-1, IL-6, TNF-alpha).

### VL Muscle Staining Procedures

Staining procedures for Oil Red O (ORO, intramuscular lipid), picrosirius red (collagen), and CD68+ cells were performed according to previous methods, and detailed procedures can be found elsewhere^11^. For ORO and picrosirius red, stained sections were imaged at 10x magnification and analyzed using a custom MatLab program^11^. The relative staining intensity for each animal was the average across the entire cross-section of each muscle section (20–50 slides/animal). Specifically for ORO, intramyofiber lipid, external muscle cell lipid deposits, and non-muscle fat-containing cells were included in the average ORO value reported for each animal. For CD68+ immunohistochemistry, 8–10 images were randomly taken across each muscle cross-section at 20x magnification (Olympus, Japan), and cells were considered positive if simultaneously stained for DAPI and Cyanine-3.

### Tissue qPCR analysis

Samples of mid-belly frozen VL and frozen visceral adipose tissue were processed as previously described using the Tri-Spin Method^11^. Oxidative stress (iNOS), pro-inflammatory (IL-1β, COX-2, MCP-1, TNF-α, IL-6, leptin,) atrophy (MuRF-1, MAFbx/atrogin-1), and fat cell differentiation (PPARγ) markers were evaluated. Primers are listed in Supplementary Table 1. All assessments were performed in duplicate under optimal conditions that conformed to qPCR criteria.

### Statistical Analysis

All groups were compared against the chow-fed control group. Levene’s test for equality of variance was conducted on all outcomes. If significant (p ≤ 0.05), Mann-Whitney U-Tests were used to evaluate each time-point compared to chow-fed controls. If equal variances were found, Student’s t-tests were performed between each individual HFS time-points and the chow-fed control group (IBM SPSS 21, α = 0.05). To understand systemic contributors to intramuscular fat and fibrosis, Pearson and Spearman correlations were run between body fat, all serum markers, all adipose tissue mRNA marker levels, all VL mRNA marker levels, VL intramuscular fat, and VL fibrosis across all animals.

## Additional Information

**How to cite this article:** Collins, K. H. *et al.* A High-Fat High-Sucrose Diet Rapidly Alters Muscle Integrity, Inflammation and Gut Microbiota in Male Rats. *Sci. Rep.* **6**, 37278; doi: 10.1038/srep37278 (2016).

**Publisher’s note:** Springer Nature remains neutral with regard to jurisdictional claims in published maps and institutional affiliations.

## Supplementary Material

Supplementary Information

## Figures and Tables

**Table 1:** VL and Visceral Adipose Tissue mRNA levels; Data are shown as Fold change compared to chow-fed controls (Mean ± SE).

mRNA levels	Vastus Lateralis				Visceral Adipose Tissue			
	3-days	7-days	14-days	28-days	3-days	7-days	14-days	28-days
	Fold Change (Mean ± SE).				Fold Change (Mean ± SE).			
iNOS	2.00 ± 0.33*	2.08 ± 0.36**	1.39 ± 0.56	1.67 ± 0.37	**1.68 ± 0.33**^**‡**^	1.74 ± 0.36***	**1.17 ± 0.56**^**‡**^	1.40 ± 0.37
COX-2	1.21 ± 0.16	1.49 ± 0.15**	1.07 ± 0.19	1.21 ± 0.13	1.96 ± 0.86	1.18 ± 0.27	0.82 ± 0.25	0.80 ± 0.18
PPARγ	2.06 ± 0.57	1.78 ± 0.14 **	1.34 ± 0.34	1.89 ± 0.41 *	1.89 ± 0.68	2.07 ± 1.01	4.10 ± 2.79	1.91 ± 0.27**
MCP-1	1.21 ± 0.23	1.43 ± 0.12*	1.35 ± 0.28	**1.62 ± 0.32‡**	1.89 ± 0.68	2.07 ± 1.01	4.40 ± 2.79	1.91 ± 0.18**
MuRF-1	1.44 ± 0.29	1.70 ± 0.18**	1.35 ± 0.26	1.62 ± 0.22*	ND	ND	ND	ND
MAFbx/atrogin-1	1.40 ± 0.33	0.73 ± 0.15	0.64 ± 0.16	0.78 ± 0.17	ND	ND	ND	ND
TNF-α	1.52 ± 0.04	1.35 ± 0.37	1.02 ± 0.49	0.77 ± 0.17	ND	ND	ND	ND
IL-6	1.13 ± 0.30	1.22 ± 0.23	1.11 ± 0.27	1.16 ± 0.20	ND	ND	ND	ND
Leptin	4.11 ± 2.40	0.78 ± 0.21	0.96 ± 0.37	12.71 ± 11.54	0.88 ± 0.36	0.81 ± 0.20	0.80 ± 0.18	0.75 ± 0.21

^‡^indicates p < 0.10 vs chow-fed control.

*indicates p < 0.05 vs chow-fed control.

**indicates p < 0.01 vs chow-fed control.

***p < 0.001 vs chow-fed control —.

ND: Not done.

**Table 2:** Serum metabolic marker concentrations.

Marker	Days on HFS				
	Chow-fed Control Mean ± SE	3 Mean ± SE	7 Mean ± SE	14 Mean ± SE	28 Mean ± SE
Glucose (mmol/L)	5.20 ± 0.13	5.68 ± 0.81	6.45 ± 0.96	5.40 ± 1.05	5.13 ± 0.88
Insulin (ng/mL)	1.41 ± 0.28	1.98 ± 0.36	1.87 ± 0.50	1.98 ± 0.36	2.28 ± 0.68

**Table 3:** Animal Ages at Start, Sacrifice, and Difference from Chow-fed Control Animals at Sacrifice.

Group	Age at start (in days) Mean ± SEM	Age at sacrifice (in days) Mean ± SEM	Difference in average age from chow at sacrifice (in days) Mean ± SEM
Chow-fed controls	74 ± 2	102 ± 2	
3 Days HFS	89 ± 2	92 ± 2	−10 days
7 Days HFS	87 ± 2	94 ± 2	−8 days
14 Days HFS	87 ± 3	100 ± 2	−2 days
28 Days HFS	87 ± 2	115 ± 2	+13 days
