# Supplementary material for: A High-Fat High-Sucrose Diet Rapidly Alters Muscle Integrity, Inflammation and Gut Microbiota in Male Rats
Source: Sci Rep. 2016 Nov 17;6:37278. doi: 10.1038/srep37278 (PMC5112513; doi:10.1038/srep37278)
Supplement: Supplementary Information [file srep37278-s1.doc]

**A High-Fat High-Sucrose Diet Rapidly Alters Muscle Integrity, Inflammation and Gut Microbiota in Male Rats**

**Kelsey H. Collins, B.S.^1,2,‡^** [khmcolli@ucalgary.ca](mailto:khmcolli@ucalgary.ca)

**Heather A. Paul, B.Sc. ^1,3^ haskochy@ucalgary.ca**

**David A. Hart, Ph.D.^2,4^** hartd@ucalgary.ca

**Raylene A. Reimer, Ph.D.^1,3^ reimer@ucalgary.ca**

**Ian C. Smith^1^** icsmith@ucalgary.ca

**Jaqueline L. Rios^1^** jaquelinelourdes.rio@ucalgary.ca

**Ruth A. Seerattan Ph.D. ^1^** rseerattan@kin.ucalgary.ca

**Walter Herzog, Ph.D.^1,2^** wherzog@ucalgary.ca

Supplemental table 1: Primer Sequences for Muscle RT-qPCR

| Primer | Forward Sequence | Reverse Sequence | Origin |
| --- | --- | --- | --- |
| COX-2 | CAG TAC ACT ACA TCC TGA CC | CGT CAA CAC GTA TCT CAT GG | S67722 |
| IL-6 | TCA CAG AAG GAG TGG CTA AG | ACC ACA GTG AGG AAT GTC CA | NM_012589 |
| iNOS | AAG GCA CAA GAC TCT GAC AC | GGA TCG CAC TTC TGT CTC TC | AB250951 |
| Leptin | CCT GTG GCT TTG GTC CTA TCT G | CTG CTC AAA GCC ACC ACC TCT G | NM_013076 |
| MAFbx/atrogin-1 | CAG CCT GAA CTA CGA TGT TG | ATG GCG CTC CTT AGT ACT CC | AY059628 |
| MCP-1 | ACT ATG CAG GTC TCT GTC AC | TGC CAG TGA ATG AGT AGC AG | M54771 |
| MuRF-1 | ATC ACT CAG GAG CAG GAG GA | CTT GGC ACT CAA GAG GAA GG | NM_080903 |
| PPARϒ | CTT GGC CAT ATT TAT AGC TGT CAT TAT T | TGT CCT CGA TGG GCT TCA C | NM_013124 |
| TNF-a | CAC GCT CTT CTG TCT ACT GA | GGC CAT GGA ACT GAT GAG AG | X66539 |
